# Reducing the Mortality Gap in People With Severe Mental Disorders: The Role of Lifestyle Psychosocial Interventions

**DOI:** 10.3389/fpsyt.2018.00463

**Published:** 2018-09-28

**Authors:** Sarah Barber, Graham Thornicroft

**Affiliations:** Centre for Global Mental Health, Institute of Psychiatry, Psychology and Neuroscience, King's College London, London, United Kingdom

**Keywords:** excess mortality, physical health, severe mental disorders, schizophrenia, bipolar disorder, depression, lifestyle psychosocial interventions, low-and-middle income countries

## Abstract

This mini-review considers the mortality gap in persons with severe mental disorder (SMD) globally. Current estimates of 10–20 years of potential life lost may be too conservative, both in high (HIC) and low and middle-income countries (LMICs). There is an emerging consensus that natural causes account for the majority of deaths in persons with SMD in both resource settings. In HICs, cardiovascular causes predominate, and can be attributed to individual, health-system and societal level risk factors. Psychosocial lifestyle interventions target behavior specific risk factors for physical ill-health. There is good evidence for tailored weight-loss programmes, but mixed evidence for smoking cessation, substance misuse and risky sexual behavior. In terms of supporting persons with SMD, nurse-led services and the utilization of peer-support are showing promise. Future research efforts must focus on effective interventions and health system models for both high and low resource settings to address this startling health inequality.

This mini-review considers the evidence for the mortality disparity between persons with severe mental disorder (SMD) and the general population worldwide, where SMD is defined as schizophrenia and other psychotic disorders, bipolar disorder and severe depression. We will explore the causes of death in high income (HIC) and low and middle-income (LMIC) countries and review the multi-level risk factor model for mortality in SMD proposed by the World Health Organization (WHO) (1). Then, focusing on behavioral risk factors, we will discuss the emerging evidence base for lifestyle psychosocial interventions. Finally, we will consider different models of professional support systems for persons with SMD.

## Mortality as an outcome measure

The WHO estimate that 10–20 years of potential life is lost in SMD (1). This wide estimate is derived from a number of meta-analyses containing data predominantly from HICs [e.g., (2)]. Concerningly, there is new evidence from HICs that the gap is increasing (3). What is the situation in LMICs? 10 years ago there was debate as to whether some outcomes, at least for schizophrenia, may in fact be better in developing countries (4, 5). This was based largely on three cross-national epidemiological studies sponsored by the WHO [IPSS (6), DOSMeD (7), ISOS (8)], which appeared to demonstrated higher rates of complete remission and better social functioning and mortality rates (expressed in standardized mortality ratios). However in a recent high quality Ethiopian study, the average potential years of life lost for persons with SMD was 28.4 years (9). This is in keeping with other cohorts in Madras (10) and Bali (11) which suggest mortality rates in LMICs may be in fact be worse.

## Causes of death in severe mental disorder

Our understanding of the causes of premature death in SMD has undergone radical change in recent decades. Historical data focusing on inpatient populations tended to over-estimate the rate of unnatural deaths in SMD (suicide, but also accidents and homicides). We now know that the mortality gap cannot be attributed to this alone. A retrospective study in Australia which linked data from mental and physical health services reported that the excess deaths were attributable to physical illnesses in over three-quarters of cases (3). This is in keeping with data from other HICs, where meta-analyses have identified cardiovascular diseases as the major cause of death in persons with SMD (12). The limited data from LMICs also suggest that the majority of deaths are due to natural illness (9–11). However, reflecting the pattern in the general population, infectious disease was the primary cause of death in an Ethiopian study (9).

Unnatural causes still account for about a quarter of deaths in persons with both in HICs and LMIC. This includes suicide, for which there is an increased risk amongst persons with SMD, especially within the first few years after contact with a psychiatric service (9, 13), but also accidental death (more common than suicide) and homicide (14, 15).

## Risk factors for excess mortality

The WHO have published a multilevel model for risk of excess mortality in persons with SMD (1). It considers individual factors (which can be disorder- or behavior-specific), health systems (such as financing) and social determinants of health (such as culture and societal values), highlighting the complexity of this problem.

Taking cardiovascular ill-health as an example, the contributing factors in persons with SMD may include disease-specific pathogenic mechanisms (see the emerging stress and inflammation theories), the metabolic side effects of antipsychotics [higher doses have been associated with higher risk of coronary heart disease and stroke (16)], behavioral factors [persons with SMD, when compared to general population controls, were less likely to be non-smokers or exercise to daily recommendations, and had poorer health outcomes (17)] and health system factors [persons with SMD are less likely to be referred for coronary revascularization procedures after a heart attack (18)] which are entwined with societal values.

Researchers in LMICs have called for further studies of the mechanisms underlying death from natural causes in persons with SMD (9). No doubt there will be identifiable risks at every level of the WHO model.

## The role of psychosocial interventions in reducing excess mortality

To reduce the mortality gap in persons with SMD, interventions are required at the individual to societal level. A full review of all of the current evidence is beyond the scope of this mini-review. We will focus on the psychosocial interventions aiming to tackle individual, behavior-specific risk factors.

Persons with SMD want to quit smoking (19), and there is some evidence of a modest benefit of smoking cessation counseling delivered by psychiatrists: number of cigarettes smoked in a typical week was significantly reduced after 12 months, however rates of abstinence determined by expired carbon monoxide was unchanged (20). There is better evidence for weight-loss programmes. Persons with SMD randomized to a tailored group weight management education and exercise sessions have achieved significant weight loss (21, 22) as well as reduced fasting glucose and medical hospitalizations after 6 months (22). Interventions aiming to reduce substance misuse and risky sexual behavior have had mixed results. One study demonstrated that an enhanced service delivered at the site of mental health treatment significantly improved testing and immunization for blood-borne infection, but despite risk reduction counseling (the psychosocial element) led to no change in risk behavior (23).

The evidence base for lifestyle psychosocial interventions in persons with SMD is mixed in both quality and results. We did not identify any studies in LMICs, where it is likely that interventions to reduce risk behavior for blood-borne infections would be highly relevant.

## Models of support

In parallel with studies addressing specific risk behaviors, different models for supporting persons with SMD to achieve good physical health are being tested. There is evidence that nurse led services, both as care managers (prompting general and specialist services) and as practioners, can increase screening rates for cardiovascular risk factors (24, 25). Further studies will be required to determine if such enhanced services for persons with SMD result in reduced mortality. Another area of promise is peer-led intervention. In the United States, a chronic disease self-management program, adapted for persons with SMD and delivered by peers led to improvements in self-rated physical and mental health scores (24). Importantly there was a clinically significant improvement in “patient activation” a measure of “individual's perceived ability to manage his or her illness and health behaviors.”

## Conclusions

In conclusion, persons with SMD are dying younger than the general population globally. There is an emerging consensus that the majority of excess mortality is due to poor physical health, with cardiovascular disease the major cause of death in HICs. Risk factor levels range from the individual (disorder and behavior specific) to the health system and social determinants. Studies have demonstrated that lifestyle psychosocial interventions have the potential to benefit persons with SMD, through tobacco-smoking cessation, increased activity and weight loss. Persons with SMD may require extra support to achieve healthy lifestyles, and both nurse-led and peer-led interventions have shown promise. However, the current evidence base for lifestyle psychosocial interventions is limited to HICs. With recent evidence demonstrating that the mortality gap may be even higher for persons with SMD in LMICs, this must become a focus for global health research.

## Author contributions

SB drafted the manuscript under the supervision of GT, who revised the manuscript.

### Conflict of interest statement

The authors declare that the research was conducted in the absence of any commercial or financial relationships that could be construed as a potential conflict of interest.
